# Low Morbidity Anatomical Revascularisation for an Infected Aorto-Bifemoral Graft Using a Staged Hybrid Procedure

**DOI:** 10.7759/cureus.36805

**Published:** 2023-03-28

**Authors:** Joel Ding, Sam Farah, Elie Haddad, Graeme C Last, Yahya Lahham

**Affiliations:** 1 Department of Vascular Surgery, Austin Health, Melbourne, AUS; 2 Department of Vascular Surgery, Peninsula Health, Melbourne, AUS

**Keywords:** explant, prosthetic infection, post-operative morbidity, bovine pericardial patch, stent graft infection, aortoiliac disease

## Abstract

A 66-year-old woman presented with a right femoral false aneurysm following an aortobifemoral bypass for lifestyle-limiting claudication. A computed tomography (CT) angiogram revealed features of complete aortobifemoral graft infection. A two-stage procedure was performed. The first hybrid stage involved the excision of only the femoral components and covered stenting of the aortic stump, along with recanalization of bilateral native iliac systems. The second stage, six weeks later, involved aortic stent and graft explant via midline laparotomy with aortic bovine pericardium patch repair (LeMaitre Vascular Inc, Burlington, Massachusetts). Follow-up imaging demonstrated no residual infection, and the patient remained without complication at the 12-month follow-up. This novel approach utilizes hybrid surgical techniques and modern bioprosthetic material to safely manage an infected aortobifemoral bypass graft.

## Introduction

Prosthetic material infection following aortoiliac or aortofemoral reconstruction is one of the most challenging complications in vascular surgery [1]. Extra-anatomic bypass with excision of infected material has traditionally been the gold standard approach [2]. However, extra-anatomic bypass, usually by axillobifemoral bypass grafting, is frequently associated with recurrent infection, as well as high morbidity and mortality [3].

We present a previously undescribed technique that utilizes modern bioprosthetic material and hybrid surgical techniques to provide low morbidity lower limb revascularisation and removal of infected prostheses. 

## Case presentation

**Figure 1:**
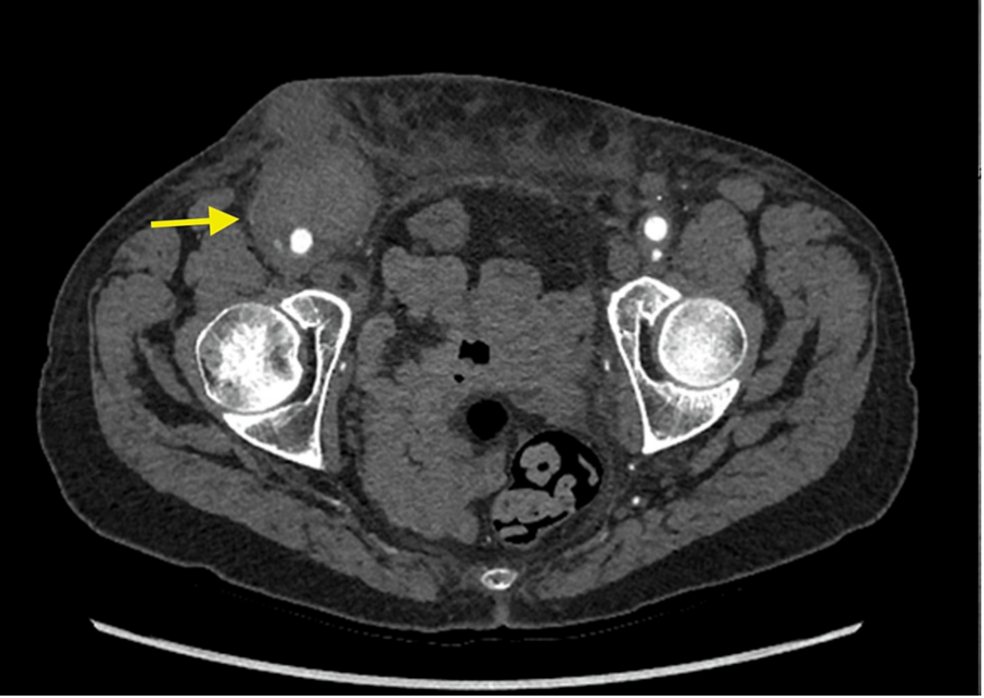
CT angiogram demonstrating a right femoral false aneurysm with surrounding suppuration

A 66-year-old woman presented with fevers and a painful indurated right femoral mass following elective aortobifemoral bypass grafting for lifestyle-limiting claudication performed 18 months prior. At the time of presentation, she was free from claudication. A computed tomography (CT) angiogram demonstrated a large right femoral false aneurysm as well as fluid collection along the entire length of the graft (Figure 1). The patient was a 40-pack-year ex-smoker and was immunosuppressed on ruxolitinib for myelofibrosis. Her vascular surgical history included a previous balloon-expandable bare metal right common iliac stent, which was chronically occluded, and bilateral patent autogenous femoropopliteal bypasses. 

Despite numerous blood and tissue cultures, no pathogen was definitively isolated, and the patient was placed on intravenous piperacillin-tazobactam 4.5g eight-hourly for a total of eight weeks. 

We planned a two-stage revascularisation procedure. The first stage involved the surgical removal of infected femoral graft components to allow the majority of suppuration to be drained. The common femoral anastomoses were patched with the great saphenous vein bilaterally, and 10 to 15cm of existing Dacron graft limb was excised from each side. Significant purulence was found encasing the right Dacron graft limb, indicating Szilagyi grade III infection. Both external and common iliac arteries were then recanalized. Following the restoration of in-line iliac flow, the aortic anastomosis was prophylactically covered with a 10mm Advanta V12 stent (Atrium Medical Corporation, Merrimack, New Hampshire) to prevent potential aortic stump "blow out" at a later stage. Two large bore corrugated drains were placed in each groin and removed one week post-operatively following no further drain output. 

Repeat CT angiography performed one week post-operatively demonstrated no infective recollection surrounding both common femoral arteries and no aortic false aneurysm. A moderate aortic collection was still present; however, its appearance was unchanged from the initial pre-operative CT. The patient was discharged on postoperative day six.

**Figure 2:**
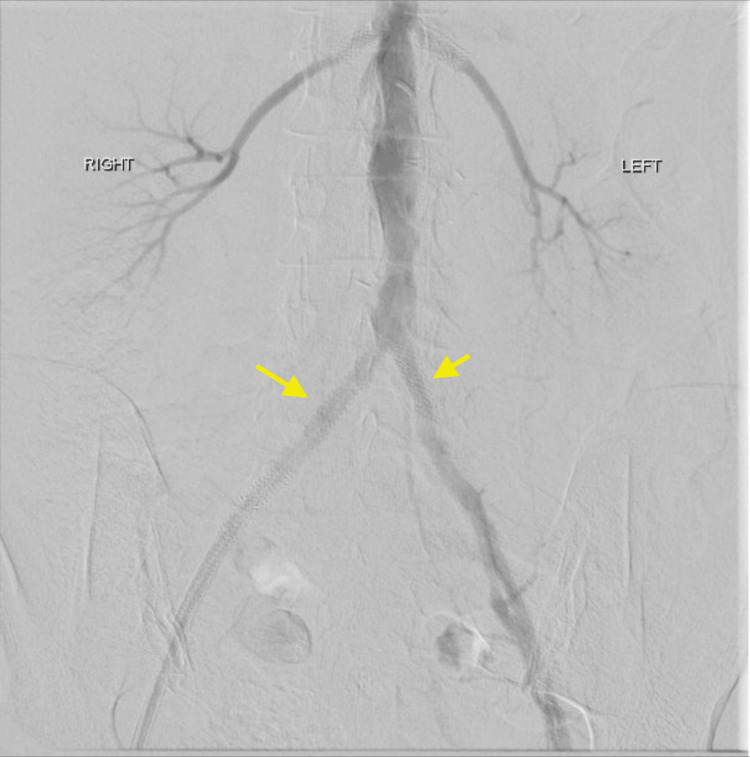
Completion digital subtraction angiogram showing bilateral patent native iliac systems, following second stage aortic graft excision and patch repair

Six weeks following the initial procedure, the patient underwent removal of the final infected aortic graft. This was performed via a midline laparotomy. A moderate amount of suppuration was identified surrounding the remaining aortic graft component. The graft was excised in its entirety, and following an explant of the aortic V12 stent, the aortic anastomotic defect was closed with a Xenosure® bovine pericardium patch (LeMaitre Vascular Inc, Burlington, Massachusetts). Completion of digital subtraction angiography (DSA) demonstrated a patent aortoiliac system with no complications (Figure 2). The patient was then discharged on postoperative day fourteen. Follow-up imaging at three and 12 months demonstrated no residual infection or false aneurysm. 

## Discussion

The management of an infected aortobifemoral prosthetic graft is one of the most challenging problems facing vascular surgeons. Traditional management of infected aortobifemoral grafts requires graft explant and extra-anatomic bypass, typically by axillobifemoral bypass grafting [3]. High morbidity has been seen with axillofemoral bypasses with a mortality of up to 30% in the early postoperative period [3-5] and also associated with a high degree of failure, graft occlusion, and limb loss [6]. Other documented strategies include staged revascularisation [3, 4], neo-aortic reconstruction with femoral vein [4], and sometimes graft retention with long-term antibiotic therapy [7]. 

The method described in this case report represents a novel use of hybrid techniques to maintain lower limb perfusion while negating the need for an extra-anatomic bypass. In the case of our immunosuppressed patient, avoiding axillobifemoral bypasses was ideal for minimizing the use of further prosthetic material, while our staged approach also allowed time for drainage and resolution of infection prior to the insertion of the bovine pericardium patch. It should be noted that native racanalization may not be technically possible in all scenarios, which limits the generalizability of our approach.

The use of a bovine pericardium patch to repair aortic anastomosis has also not been previously described. Although the patient has demonstrated no recurrent infection at 12 months, the success of this case report is limited as it is unclear if the bovine pericardium patch will remain infection-free in the longer term.

## Conclusions

Infected aortofemoral grafts are one of the most feared complications facing vascular surgeons today. The advent of hybrid vascular surgery has presented new solutions to this challenging problem. Our case describes a novel approach of staged infected graft explant followed by interval in-situ endovascular reconstruction, which may be a safer alternative to traditional extra-anatomical bypass grafting. Our technique should be considered in immunosuppressed patients, where the use of further prosthetic material should be avoided. The XenoSure® bovine pericardium patch is also emerging as a biological alternative to prosthetic grafts for arterial reconstruction in infected fields. Further randomized control trials comparing bovine pericardium to an autologous vein or other prosthetic material are needed to prove their role in vascular infection.
